# Prevalence of Oropharyngeal Dysphagia in Patients With Advanced Cognitive Impairment

**DOI:** 10.1002/alz.093170

**Published:** 2025-01-09

**Authors:** Ana Carolina Borges de Miranda Souza, Lilian Schafirovits Morillo

**Affiliations:** ^1^ University of São Paulo Medical School, São Paulo, São Paulo Brazil; ^2^ University of São Paulo Medical School, São Paulo Brazil

## Abstract

**Introduction:**

Patients with severe cognitive impairment, with the progression of the disease, show behavioral impairments, loss of functionality and, in many cases, swallowing changes (dysphagia). Dysphagia comes with serious complications that can cause health damage, such as malnutrition, dehydration and serious lung damage secondary to aspirations. Eating process goes beyond nutritional intake, as it has a social, cultural, behavioral, physical and cognitive component. OBJECTIVE: To correlate swallowing changes with the severity of symptoms that are present in patients with advanced cognitive impairment, monitored by the multidisciplinary team at an University hospital in the state of São Paulo.

**Method:**

This is a longitudinal observational retrospective study, carried out through analysis of medical records of patients followed at the Advanced Cognitive Impairment Outpatient Clinic (ACCA) over a period of one year. A comparison was made between the swallowing functionality scale (ASHA NOMS) and clinical dementia rating sum of boxes score (CDR‐SB).

**Results:**

The prevalence of patients with Alzheimer’s disease was 33.3% of the sample of this population, followed by mixed dementia (Alzheimer’s and vascular). In August 2022, 27.5% of patients had ASHA 6 or 7, 23.5% had ASHA 5, 47.1% had ASHA 4 or less and 25.5% of the patients had CDR 3 (sum of boxes 17). After a year, only 11.7% had ASHA 6 or 7 (normal and/or adapted swallowing), 33.3% of patients had ASHA 5 and 49% had ASHA 4 or less (needs restrictions and adaptations of consistencies, use of strategies, food supplementation and/or use of alternative feeding routes), despite dementias progression CDR 3 (sum of boxes 18) in 31.4% of the total amount. Comparing 2022 datas of ASHA and CDR scale, an inverse proportionality relationship was found with statistical relevance (p = 0.05), but this finding did not remain in 2023 (p = 0.73).

**Conclusion:**

Oropharyngeal dysphagia is present in many cases of cognitive impairment and speech therapy assessment is essential to identify changes in swallowing, manage and make dietary adaptations aiming to maintain, for nutrition or comfort, a safe oral route, avoiding respiratory infections and, consequently, mortality.

